# High-power light-emitting diode array design and assembly for practical photodynamic therapy research

**DOI:** 10.1117/1.JBO.25.6.063811

**Published:** 2020-04-15

**Authors:** Eric M. Kercher, Kai Zhang, Matt Waguespack, Ryan T. Lang, Alejandro Olmos, Bryan Q. Spring

**Affiliations:** a Northeastern University, Translational Biophotonics Cluster, Boston, Massachusetts, United States; b Northeastern University, Department of Physics, Boston, Massachusetts, United States; c Northeastern University, Department of Health Sciences, Boston, Massachusetts, United States; d Northeastern University, Department of Bioengineering, Boston, Massachusetts, United States

**Keywords:** light-emitting diodes, photodynamic therapy, aminolevulinic acid, benzoporphyrin derivative, verteporfin

## Abstract

**Significance:** Commercial lasers, lamps, and light-emitting diode (LED) light sources have stimulated the clinical translation of photodynamic therapy (PDT). Yet, the continued exploration of new photosensitizers (PSs) for PDT often requires separate activation wavelengths for each agent being investigated. Customized light sources for such research frequently come at significant financial or technical cost, especially when compounded over many agents and wavelengths.

**Aim:** LEDs offer potential as a cost-effective tool for new PS and multi-PS photodynamic research. A low-cost-per-wavelength tool leveraging high-power LEDs to facilitate efficient and versatile research is needed to further accelerate research in the field.

**Approach:** We developed and validated a high-power LED array system for benchtop PDT with a modular design for efficient switching between wavelengths that overcome many challenges in light source design. We describe the assembly of a low-cost LED module plus the supporting infrastructure, software, and protocols to streamline typical *in vitro* PDT experimentation.

**Results:** The LED array system is stable at intensities in excess of $100\;\mathrm{mW}/{\mathrm{cm}}^{2}$ with 2.3% variation across the illumination field, competitive with other custom and commercial devices. To demonstrate efficacy and versatility, a primary ovarian cancer cell line was treated with two widely used PSs, aminolevulinic acid and verteporfin, using the LED modules at a clinically relevant $50\;J/{\mathrm{cm}}^{2}$ light dose that induced over 90% cell death for each treatment.

**Conclusions:** Our work provides the community with a tool for new PS and multi-PS benchtop photodynamic research that, unlike most commercial light sources, affords the user a low barrier to entry and low-cost-per-wavelength with the goal of illuminating new insights at the forefront of PDT.

## 1 Introduction

The basis of photodynamic therapy (PDT) involves the induction of cytotoxicity—typically via the creation of intracellular reactive oxygen species—through a mediating chemical, or photosensitizer (PS).^1^ Critically, toxicity is induced specifically through absorption of nonionizing electromagnetic radiation by the PS, such that two ingredients, having minimal side effects by themselves, are only harmful when combined. This phototoxic effect, and many variations thereon, have been exploited to treat various dermatological,^2^ oncological,^3^ and infectious diseases.^4^ Excellent descriptions of the physical and biochemical processes behind PDT,^5^^–^^7^ as well as many reviews and historical accounts of the field,^1^^,^^3^^,^^8^^–^^12^ may be found elsewhere in the literature.

The discussion of light sources and delivery methods in such reviews is often superseded by noteworthy chemical, physical, and biological discoveries and insights regarding PS development and clinical efficacy (with some exceptions^7^^,^^13^). Despite the frequent lack of emphasis, light delivery itself is widely appreciated as a fundamental component of photodynamic research and is critical to the clinical success of past and future therapies. For example, the invention of the helium-neon laser (632.8 nm) in 1962^14^ enabled Dougherty and colleagues to complete some of the first clinical studies on hematoporphyrin derivative in 1978,^15^ thereby paving the way for Photofrin^®^ and other porphyrin-based PSs to be investigated and translated. Since then, the continued development of new light sources has accelerated the development of new PSs and applications for clinical PDT.^10^

Presently, light sources used for photoactivation can be organized into three categories.^7^^,^^13^ First and most widely implemented are lasers, which are desirable for their efficient, high-power, and coherent output. Some laser cavity designs used for PDT include argon dye, Cu- and Au-vapor, frequency-doubled Nd:YAG, solid-state, and semiconductor diodes.^13^ Lasers also generally offer the highest density of photons for fiber coupling and endoscopic or interstitial light delivery. Second, filtered lamps provide relatively uniform wide-field illumination but have not found much use outside of dermatological settings.^13^ Finally, light-emitting diodes (LEDs) disperse incoherent light from a small semiconductor with an intensity proportional to the current across the diode. They offer a compromise between lasers and lamps and can reach intensities above 1 W.^16^ LEDs are also versatile when assembled into linear arrays for endoscopic light delivery or two-dimensional arrays, which can illuminate broad areas comparable to most lamps.^7^^,^^13^

Developing a phototherapeutic strategy for a given disease requires the right combination of PS pharmacokinetics and localization, light source, light dose, and drug-to-light interval.^9^ These parameters depend largely on the disease morphology and pathology but also on economic, regulatory, and epidemiological factors regarding the patient population. In addition, financial and commercial considerations that arise during clinical translation can be difficult to navigate.^17^ Ultimately, a unique light source is often required to accompany each PS under different applications. To illustrate this, three FDA-approved agents—all approved using different light sources for photoactivation—serve as informative examples.

First, aminolevulinic acid (ALA, Levulan^®^), approved in 1999 for treatment of mild to moderate actinic keratosis (AK), is activated using a blue fluorescent lamp (BLU-U^®^ Blue Light Photodynamic Therapy Illuminator) at 417±5  nm wavelength with a recommended dose of $10\;{J/\mathrm{cm}}^{2}$ over 16 min and 40 s.^18^ Second, methyl aminolevulinate (MAL, Metvixia^®^) was FDA approved for AK in 2004 paired with a metal halogen lamp at 570 to 670 nm (CureLight Broadband, Model CureLight 01). Based on prior preclinical data,^19^ a follow-up clinical trial in 2008 (NCT00304239) demonstrated LED illumination at 630±5  nm (Aktilite^®^ CL128) to be a more effective source for MAL activation. In so doing, the recommended light dose was decreased from $75\;{J/\mathrm{cm}}^{2}$ over 8 to 12 min using the broadband source to $37\;{J/\mathrm{cm}}^{2}$ over 7 to 10 min using the LED array, consistent with others’ findings.^20^^,^^21^ Although effective, treatment of AKs and nonmelanoma skin cancers with topical ALA/MAL requires a relatively long treatment time and causes a moderate to severe burning sensation in many patients.^22^

Third, benzoporphyrin derivative (BPD or verteporfin, Visudyne^®^), approved in 2000 to treat the wet form of age-related macular degeneration (AMD), is activated via laser at 689±3  nm. The recommended dosing strategy is $50\;{J/\mathrm{cm}}^{2}$ at $600\;{\mathrm{mW}/\mathrm{cm}}^{2}$ over 1 min and 23 s,^23^ in stark contrast with lower intensity AK treatment guidelines. The differences in light source and dosing strategies between ALA/MAL and verteporfin are simply explained by the physical differences in disease presentation. Despite other side effects, verteporfin PDT in the eye avoids sensory nerves responsible for pain, allowing for a much higher light intensity. In addition, the size of the treatment area (abnormal choroidal neovasculature that grows into the macula) is much smaller than typical AK lesions. These and other factors make a laser source ideal for treatment of AMD, whereas an LED array is more suited for large and/or disperse dermatological indications.

Lasers, despite the advantages mentioned, present a formidable cost per wavelength and require special safety equipment and protocols during use. Additionally, in cases where a direct comparison is possible, researchers have found LEDs and lasers do not differ in their treatment efficacy,^20^^,^^24^^–^^28^ whereas others have shown LEDs are just as effective as lamps^19^^,^^26^^,^^28^ and more effective than sunlight.^29^ Furthermore, LEDs are now widely used to treat many dermatological diseases,^30^^,^^31^ and a growing body of work suggests LEDs will have a significant role to play in the future of PDT.

However, excitement around the use of LEDs for PDT has been stymied by financial and technical hurdles. Existing LED-PDT sources remain expensive and caution should be taken when employing these devices.^32^ Furthermore, reports of clever adaptations of nonclinical LED sources for PDT, including dental curing lights,^16^^,^^33^ traffic lamps,^34^ and lighting fixtures,^26^^,^^35^ suggest a lack of variety, versatility, or accessibility in available clinical LED sources. This may be explained, at least in part, by a lack of competition and limited market size that has discouraged an exciting and robust industry. Those with more specific irradiation requirements, and the appropriate motivation and funding, have reported custom-assembled LED sources for various applications,^27^^,^^36^^–^^40^ albeit with limited output powers that require tedious experimental protocols. A light source or system that is cost-effective and generalizable to multiple PSs has not yet been reported.

Here, we introduce a protocol for custom LED array assembly and supporting infrastructure for cost-effective and versatile PDT research that considers and overcomes many challenges in LED array design. The device is capable of output powers in excess of $100\;\mathrm{mW}/{\mathrm{cm}}^{2}$, competitive with most commercial LED and laser sources, and a modular design enables easy switching of wavelengths for research with different PSs. The infrastructure surrounding the module is designed to streamline the experimentation process and allows the user to monitor the LED temperature in real time using a custom software interface and data acquisition (DAQ) card. The viability of these features is demonstrated via treatment of monolayer cell cultures using both ALA- and BPD-PDT. This work improves on a growing trend of LED-PDT research and will aid current and future laboratories in their photodynamic research, especially with the emergence of next-generation PSs.

## 2 Materials and Methods

### 2.1 LED Module Design and Assembly

The LED module is designed around an aluminum-substrate printed circuit board (PCB) to maximize heat flow away from the LEDs. This design choice constrains the selection of electronics to only surface-mounted components. Circuits were designed to connect four rows of four 690-nm LEDs (1 W Infrared LED, Shenzen Fedy Technology Co.) or six 635-nm LEDs (PLR3535AA000, Plessy Semiconductors) in parallel with 45 W, 10  Ω current limiting resistors (TKH45P10R0FE-TR, Ohmite). A thermistor [B57452V5103J062, Epcos (TDK)] and a 499  Ω resistor (RNCP0805FTD499R, Stackpole Electronics Inc.) circuit were also included, with the thermistor placed in the center of the LED array to enable real-time measurements of the board temperature. The full circuit required an 8-pin connector that was made from a Dupont connector kit (WYTP07-KIT, WayinTop). The complete circuit board was designed using electronic AutoCAD software (Eagle, Autodesk) and manufactured by a PCB fabrication service (PCBCart).

**Fig. 1:**
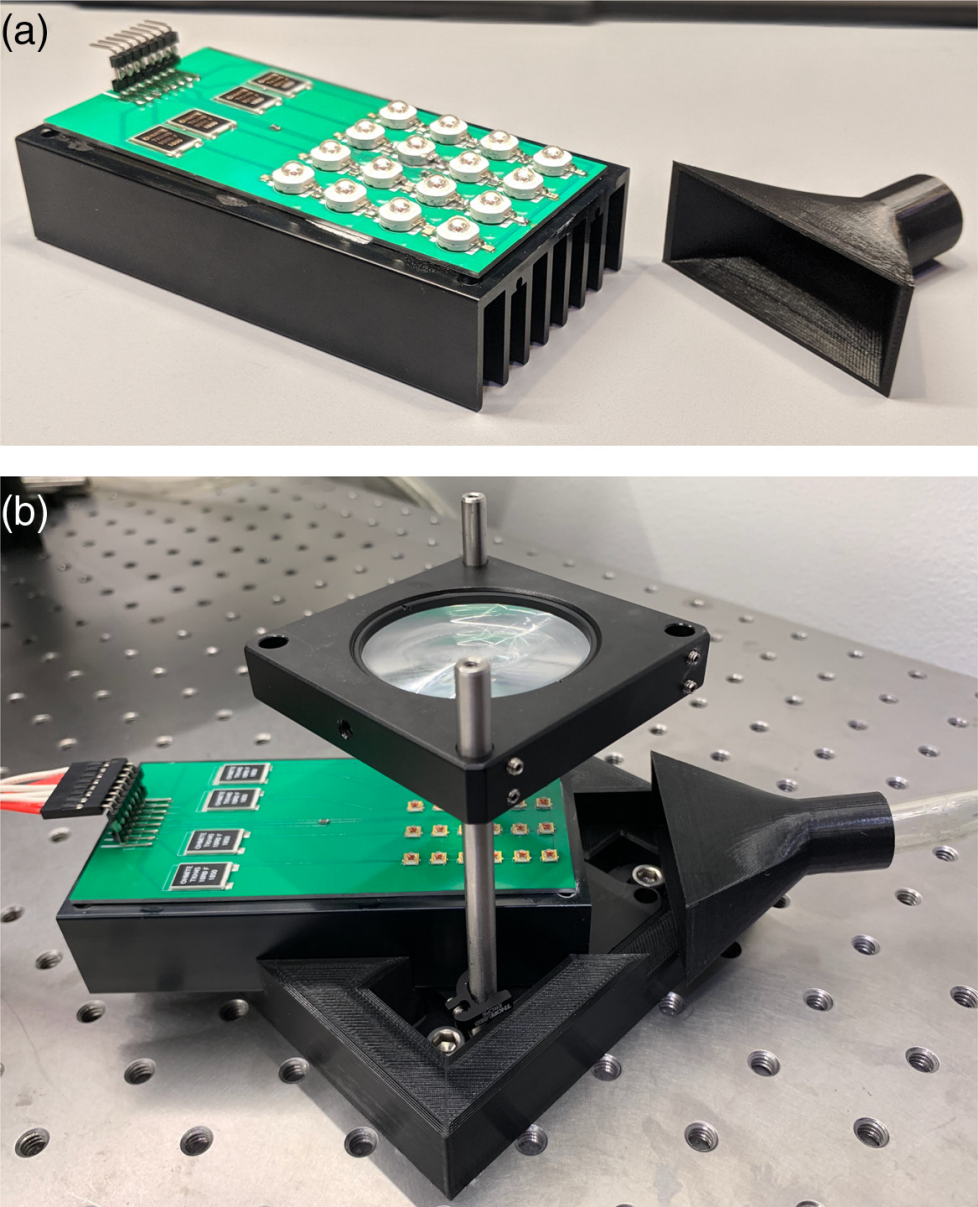
Custom-designed, high-power, modular LED array for PDT. (a) A 690-nm LED module aside a 3D-printed nozzle for air cooling. (b) A 635-nm LED module mounted on a 3D-printed brace with focusing optic above. A plexiglass surface with an aperture (not shown) is secured directly above the lens on which a well plate is placed for PDT experimentation.

To assemble the board, the components were applied to the PCB via solder paste (EP256, Kester) and, after all the components were added, a hot air gun (898D Soldering Station, Vivohome) set to 400°C was used to solder each component in place. To do this, the PCB was supported with a third hand (01902, Neiko) and the hot air gun was directed at the PCB from 3 to 4 cm below. A digital multimeter (DMM, WH5000A, AstroAI) was used to confirm that successful connections were achieved. The PCB was then mated to a heat sink (M-B012, Cincon) with a thin layer of thermal paste (Protronix-PST-D Series 7, Protronix), and six drops of super glue (PR40, 3M), three on each side, were added along the sides of the PCB to secure it in place (Fig. 1).

### 2.2 Supporting Electronics and Optomechanics

The LED circuit was connected to a 30 V/5 A power supply (HY3005F-3, Dr.meter) with an intermediate single channel 5 V relay (MK1LU5V6P, Ficbox) to enable programmable LED modulation via a USB DAQ card (USB-6001, National Instruments). The relay was wired to the 5 V power supply, digital ground, and digital I/O channel on the DAQ. The thermistor circuit was also wired to the 5 V DAQ power, with differential signal measured between analog input and ground channels. A subminiature version A (SMA) coaxial connector (8541674577, Maxmoral) was augmented with signal and ground wires in order to read the analog power meter signal through the DAQ from the SMA output on the power meter. The DAQ was connected to a laptop placed next to the experimental setup via USB to USB-c cable (National Instruments). A complete wiring diagram is provided in Fig. S1 in the Supplementary Material.

The LED module was placed on the surface of an optics table via a custom 3D-printed brace (Ender 5, Comgrow) [Fig. 1(b), Fig. S2(a) in the Supplementary Material] that secures the module to a vertical cage mounting system (CPVM, Thorlabs). Alternatively, an aluminum breadboard (MB12, Thorlabs) may be used for a mobile-cart design or if an optics table is not available. A diffuser-lens pair was placed above the LED module using a 2-in. cage mount (LCP01, Thorlabs) to focus and smooth the light field. The choice of the focusing lens was determined empirically between a Fresnel lens (FRP232, Thorlabs) and an aspheric condenser lens (ACL5040U-DG6-A, Thorlabs), with a 600-grit diffuser (DG20-600, Thorlabs) mounted just below each optic. A power meter sensor (S130C, Thorlabs), connected to a compatible power meter (PM100D, Thorlabs), was placed above, but off-center to, the mounted optic. The lens was then adjusted along the z axis to maximize the power transmission and determine the height of maximum collimation (i.e., the focal point of the lens). Both lenses allowed similar power transmission, but the Fresnel lens was found to produce a more uniform light field with a larger spot size than the condenser lens and was therefore chosen for the ensuing experiments.

A platform on which to place a well plate for typical *in vitro* PDT experimentation was fashioned from a transparent plexiglass board (SL-AS6, 12×12×1/4-in., SimbaLux). The board was covered in light-blocking tape (T205-1.0, Thorlabs), except for a 3.85×3.85-cm opening in the center. Two 3/8-in. holes were drilled on the side of the board and secured to two 4-in. optical posts (TR4, Thorlabs) mounted (UPH1, Thorlabs) to the optics table using 5/8-in. screws (SH25S063, Thorlabs). This fixed the plate height at 4 in. above the table and x−y adjustments were made to align the aperture above the lens.

Since cooling of the module is paramount, we used a compressed air line to provide active cooling of the LED module. A fan could be used in place of the compressed air. Approximately 1 m of 3/8-in. tubing was connected to the air outlet valve on one end and to a 3D-printed nozzle on the other [Fig. 1, Fig. S2(b) in the Supplementary Material]. The nozzle was designed to match the cross-section of the heat sink and provide uniform airflow through the fins. It was secured to the table via two 2-in. optics posts (TR2, Thorlabs) combined with a right-angle clamp (RA90, Thorlabs) and a fixed position lens mount (NRC MH-2P, Newport) such that the air was directed through the heat sink fins.

### 2.3 Software

We employed MATLAB’s (R2019b, MathWorks) DAQ toolbox to interface with the DAQ via custom software application, ExpressPDT, written with MATLAB’s application programming software App Designer (Fig. S3 in the Supplementary Material). Note that compatibility of the DAQ toolbox restricts full operation of the software (i.e., connection to the DAQ and relay control) to Windows operating systems. However, ExpressPDT may still be used as a PDT planning tool on any operating system.

### 2.4 Thermistor Model and Calibration

A thermistor is a resistor whose resistance changes predictably with temperature. We employed this device in a simple voltage divider circuit to measure the on-board temperature of the LEDs in real time. For negative temperature coefficient thermistors, the resistance R varies exponentially with temperature T according to

$$R=R_{0}e^{\beta (\frac{1}{T}-\frac{1}{T_{0}})},$$ (1)

where $R_{0}$ is a known resistance at some temperature $T_{0}$, and β is a fundamental property of the thermistor. Grouping the constant terms together and assuming β is constant, Eq. (1) can be rewritten to show that

$$R=R_{\infty }e^{\frac{\beta }{T}},$$ (2)

where $R_{\infty }$ is the resistance of the thermistor at very high temperatures. This model is easily linearized as

$$\ln\;R=\frac{\beta }{T}+\ln\;R_{\infty }$$ (3)

and can be fit using the variables ln R and 1/T in order to extract β and $\ln\;R_{\infty }$ as slope and intercept via linear regression. The final step is to connect the thermistor resistance to the voltage measured by the DAQ. The equation describing a simple voltage dividing circuit is as follows:

$$\frac{V}{R}=\frac{V_{+}}{R+R_{s}},$$ (4)

where V and R are the thermistor voltage and resistance, respectively, $V_{+}$ is the supply voltage, and $R_{s}$ is the resistance of the static resistor. Combining Eqs. (3) and (4) and rearranging terms give the temperature (in Celsius) as a function of thermistor voltage:

$$T(V)=\frac{\beta }{\ln\;\frac{R_{s}}{R_{\infty }}(\frac{V_{+}}{V}-1)}-273.15.$$ (5)

Although β (and therefore, $R_{\infty }$) varies slightly with temperature, the LEDs are constrained to a ∼20°C operating range over which β changes by <1%, so the assumption of constant β is valid for monitoring the LED array board. We also assume a constant 5 V supply voltage $V_{+}$ and 499  Ω resistance $R_{s}$.

The parameters β and $R_{\infty }$ were determined experimentally for each LED module by measuring the thermistor voltage across a range of temperatures. First, approximately 0.2 mL of thermal paste was placed on the thermistor, and the module was refrigerated at 4°C for 2 h. The module was then reconnected to the power supply and a thermocouple connected to the DMM was inserted into the thermal paste to determine the temperature of the thermistor. Using ExpressPDT’s calibration mode, the temperature and thermistor signal were recorded simultaneously as the module returned to room temperature. The LEDs were then turned on at low power to facilitate further warming of the module at approximately 1°C every 5 to 10 s. The LEDs were turned off and recording ceased once the module reached 65°C; 40 to 50 data points were collected in total for each module. Once the calibration procedure was complete, the thermal paste was cleaned from the LED module using cotton swabs and optic wipes dampened with isopropyl alcohol. Data were linearized and fit to Eq. (3) using Prism 8 (GraphPad); best-fit values β and $R_{\infty }$ are reported with their standard errors (SE). These values were programmed into ExpressPDT for automated temperature monitoring using Eq. (5).

### 2.5 Module Characterization

The emission spectrum of each LED module was measured with a spectrometer (Amadeus AMA01338, Ocean Optics) at room temperature (21°C), and then again at 38°C to characterize the effect of temperature on the spectral emission. The LED supply voltage was then adjusted so that the power meter read $∼100\;{\mathrm{mW}/\mathrm{cm}}^{2}$ peak power at the plate surface. Once the temperature stabilized, the temperature and power were recorded for 30 min at 1-s intervals using ExpressPDT. To assess the power delivered to each well of a cell culture 24-well plate, the module was turned on and allowed to stabilize at 39°C. The power at the plate surface in each quadrant of the aperture was measured thrice with a power meter to determine the intensity given to each well in 2×2-well experimental group. Average temperature and intensity are reported as mean±standard deviation (SD) with the coefficient of variation (CV, defined as SD divided by mean) provided where useful.

### 2.6 Cell Culture and PDT

Human primary high-grade serous ovarian cancer line (Powder, Cellaria Biosciences) was cultured in T75 Flasks (1256685, Thermo Scientific) according to a protocol recommended by Cellaria Biosciences in a humidified incubator at 5% ${\mathrm{CO}}_{2}$ and 37°C. Powder cells were cultured in Renaissance essential tumor medium (RETM) and RETM supplement (CM-0001, Cellaria Biosciences) completed with 6.3% heat-inactivated fetal bovine serum (FBS, SH30071.03HI, Hyclone™ GE Healthcare Life Sciences) and 1% penicillin/streptomycin (BP295950, Fisher BioReagents).

Before plating, RETM was prepared by diluting stock media to 3% FBS and was used throughout the experiment. During passaging, cells were lifted with 0.25% trypsin ethylenediaminetetraacetic acid (25053CI, Corning), washed in phosphate-buffered saline (PBS, 70011069, Gibco), and suspended at 20,000  cells/mL in RETM at 3% serum. One mL of cell solution was added to each well of a black-walled, 24-well plate (P241.5HN, Cellvis) and allowed to grow for 3 days. PS was administered at 0.5 mM ALA (A3785, Sigma Aldrich) or 0.1  μM verteporfin (Visudyne^®^, QLT Phototherapeutics, Inc.) in media and incubated for 4.5 or 2 h, respectively. Just before illumination, all wells were aspirated and replaced with fresh media.

To increase protocol efficiency, experimental groups were organized into 2×2-well groups (four biological replicates) to be illuminated simultaneously. Six treatment groups in each 24-well plate included three controls—no $\mathrm{PS}+0\;{J/\mathrm{cm}}^{2}$, no $\mathrm{PS}+50\;{J/\mathrm{cm}}^{2}$, and $\mathrm{PS}+0\;{J/\mathrm{cm}}^{2}$—and three treatment groups—PS+10, 20, or $50\;{J/\mathrm{cm}}^{2}$. For ALA-PDT, the average irradiance of the 635-nm module in each quadrant was $86.6\;{\mathrm{mW}/\mathrm{cm}}^{2}$ at a stable module temperature of 39°C, with a total plate illumination time of 25 min. For BPD-PDT, the average irradiance of the 690-nm module in each quadrant was $79.7\;{\mathrm{mW}/\mathrm{cm}}^{2}$ at 39°C, with a total plate illumination time of 27 min and 10 s. Laser safety goggles (100-38-245, Laser Safety Industries) with optical density (OD) 2+ at >630  nm were worn during illumination. All work with PSs was done in subdued light and plates were protected from light with aluminum foil except during PDT.

Cell culture viability was assessed with fluorescent live/dead staining 24 h after light treatment using flow cytometry (FC) and validated with confocal microscopy. Three out of the four wells from each group were collected and stained with live/dead fixable green (L-34970, Life Technologies) for 30 min at 4°C protected from light. Cells in suspension were included in this analysis by collecting the supernatant before lifting the cells. After staining, samples were washed a further 2 times in PBS, resuspended in 300  μL PBS, and immediately analyzed using a flow cytometer (Attune NxT, ThermoFisher). Both the concentration and viability of cells were measured by FC; relative viability is reported here as the average±SE concentration of viable cells normalized to the no treatment control.

Immediately after three replicates from each treatment group were collected from the plate for FC analysis, the fourth well was washed with PBS (discarding the supernatant) and stained with a 1:60 dilution of acridine orange (AO)/propidium iodide (PI) (F23001, Logos Biosystems) in media and immediately imaged using a laser-scanning confocal microscope (LSM 800, Zeiss). Images are considered as a strictly qualitative assessment of viability to validate quantitative FC results.

## 3 Results

### 3.1 Thermistor Calibration

**Fig. 2:**
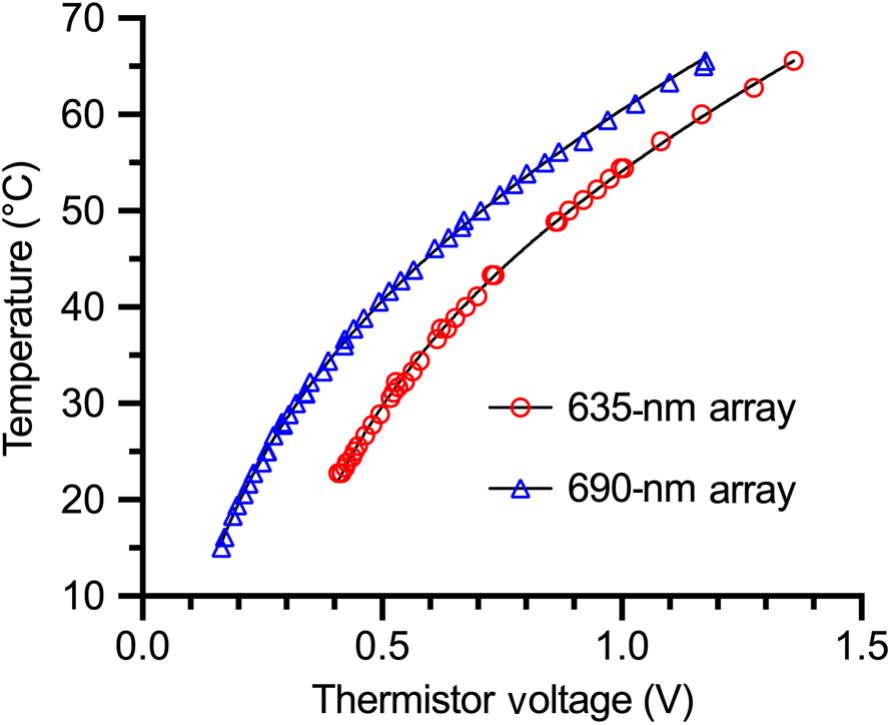
Thermistor calibration. Temperature and voltage measurements (symbols) were used to find the best-fit values for Eq. (5) (lines). For clarity, the 635-nm array data set is shifted horizontally by +0.2  V.

The thermistors on each module demonstrated ideal behavior across the range of operating temperatures (20°C to 40°C, Fig. 2). Temperature and voltage were fit to a linearized model [Eq. (3)] with slope β and intercept $R_{\infty }$ (Fig. S4 in the Supplementary Material). For the 635-nm array, we found β=4442±16  K and $R_{\infty }=3.34\pm 0.17\;m\Omega$. For the 690-nm array, we determined β=4279±7  K and $R_{\infty }=5.37\pm 0.13\;m\Omega$, in agreement with manufacturer specified values. These data were then programmed into ExpressPDT software using Eq. (5) to enable real-time module temperature monitoring during treatment.

### 3.2 LED Module Characterization

**Fig. 3:**
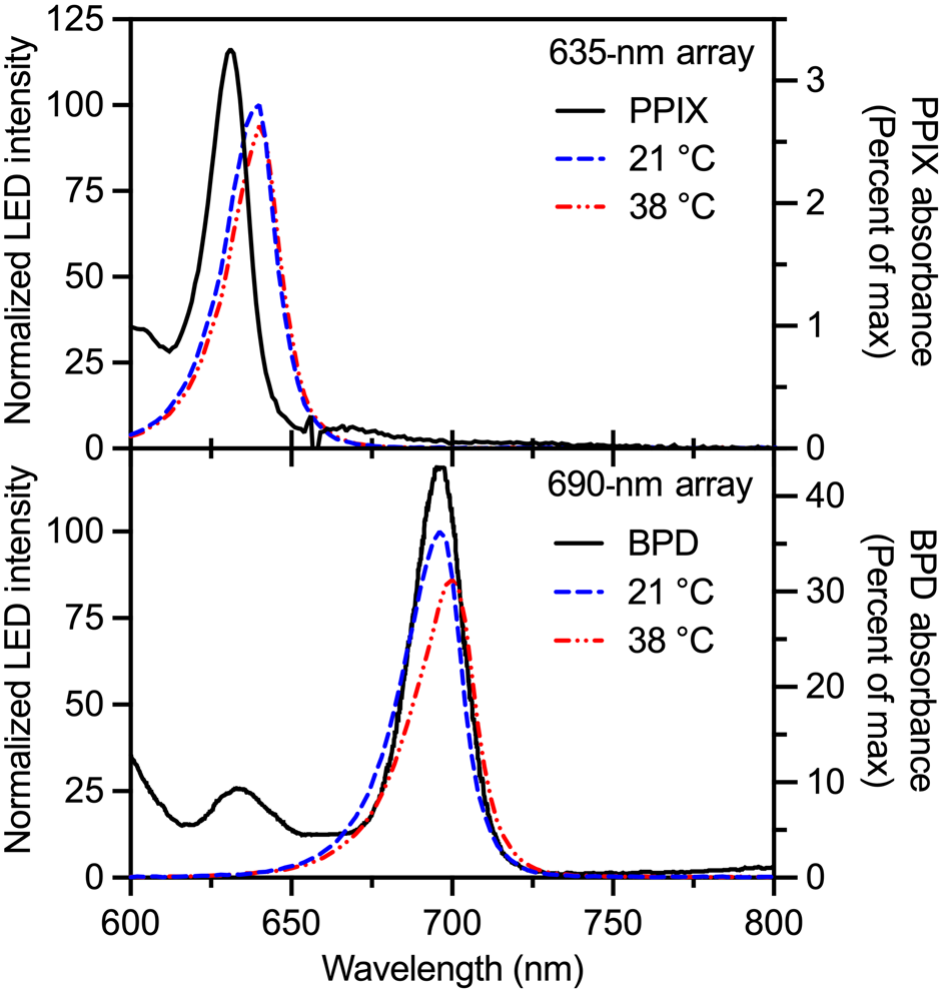
LED array spectral emission (left, y axis) at room temperature (21°C) and operating temperature (38°C) align with their respective PS absorption spectra (right, y axis). Relative intensities are preserved between low- and high-temperature spectra after normalization.

At 38°C, the LED emission underwent slight redshifting and loss of intensity compared to room temperature (Fig. 3). The 635-nm spectrum redshift was less than the spectrometer resolution (<2  nm), and the relative intensity was 94% of the room temperature spectrum. The 690-nm LED fidelity was slightly more impacted at 38°C with a redshift of 4 nm at 86% of room temperature power. These shifts were not enough to compromise the PS excitation efficiency.

**Fig. 4:**
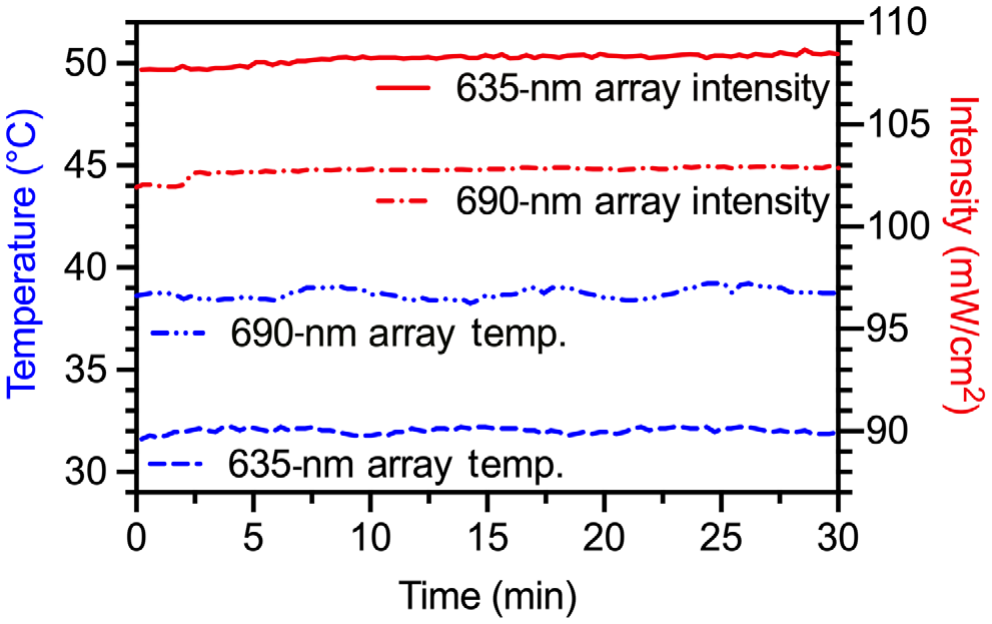
LED stability. Both LED modules are stable at operating temperature and demonstrated <1% variation in on-board temperature (left, y axis) and peak power (right, y axis) over a 30-min illumination (measurements taken at 1-s interval). Peak power was measured along the optical axis at the well-plate surface.

Thermal stability was assessed over a 30-min trial by measuring the temperature and peak power of the LED array at 1-s intervals. Both the 635- and 690-nm boards displayed <1% variation in both variables over that time frame (Fig. 4). Specifically, the 635-nm array was stable at 32.0±0.1°C (CV=0.42%) with an output of $108.2\pm 0.3\;{\mathrm{mW}/\mathrm{cm}}^{2}$ (CV=0.29%). Similarly, the 690-nm array was stable at 38.7±0.3°C (CV=0.66%) with an output of $102.8\pm 0.2\;{\mathrm{mW}/\mathrm{cm}}^{2}$ (CV=0.24%).

**Fig. 5:**
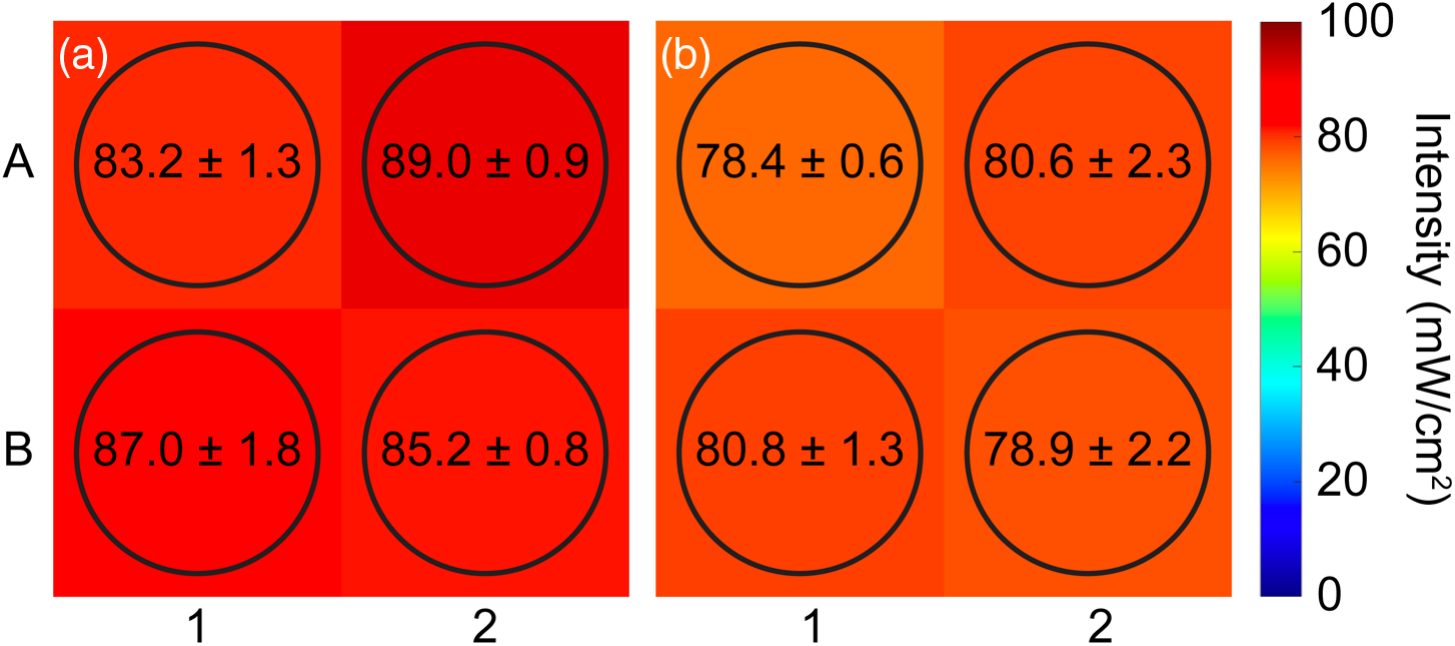
Light field assessment of the (a) 635-nm and (b) 690-nm LED modules. Intensity was measured in each quadrant of the 3.85×3.85-cm aperture to estimate the power delivered to each well of a 2×2-well experimental group (A1 and B2). Circles represent approximate location and area of the power meter sensor within each quadrant of the aperture. Results are mean±SD of 3 measurements. One-way ANOVA of the four quadrant measurements was significant for the 635-nm array (p=0.0142) and not significant for the 690-nm array (p=0.3081). Follow-up analysis with Tukey’s multiple comparisons test on the 635-nm array group revealed the intensity in well A2 was significantly larger than A1 (p=0.0207) and B2 (p=0.0207), but not different from B1 (p=0.2594). Reported p-values are adjusted for multiple comparisons.

The light field at the plate surface was analyzed for uniformity by acquiring measurements centered at the positions of each of the four wells being treated simultaneously (Fig. 5). It was determined that the variations in the power delivered to each well were 2.3% for both arrays. At operating temperature (39°C), the intensity was $86.6\pm 2.0\;{\mathrm{mW}/\mathrm{cm}}^{2}$ (CV=2.3%) for the 635-nm array and $79.7\pm 1.8\;{\mathrm{mW}/\mathrm{cm}}^{2}$ (CV=2.3%) for the 690-nm array. Individual well measurements were further analyzed to check for inhomogeneities in the light field. It was determined that for the 635-nm module, the power delivered to well A2 was $3.9\;{\mathrm{mW}/\mathrm{cm}}^{2}$ (4.3%) larger than to A1 (p=0.0207) and B2 (p=0.0207) but was no different from B1 (p=0.2594), indicating a minute nonuniformity in the photon flux through the aperture. No differences in light delivery from 690-nm module were detected (p=0.3081).

### 3.3 PDT

**Fig. 6:**
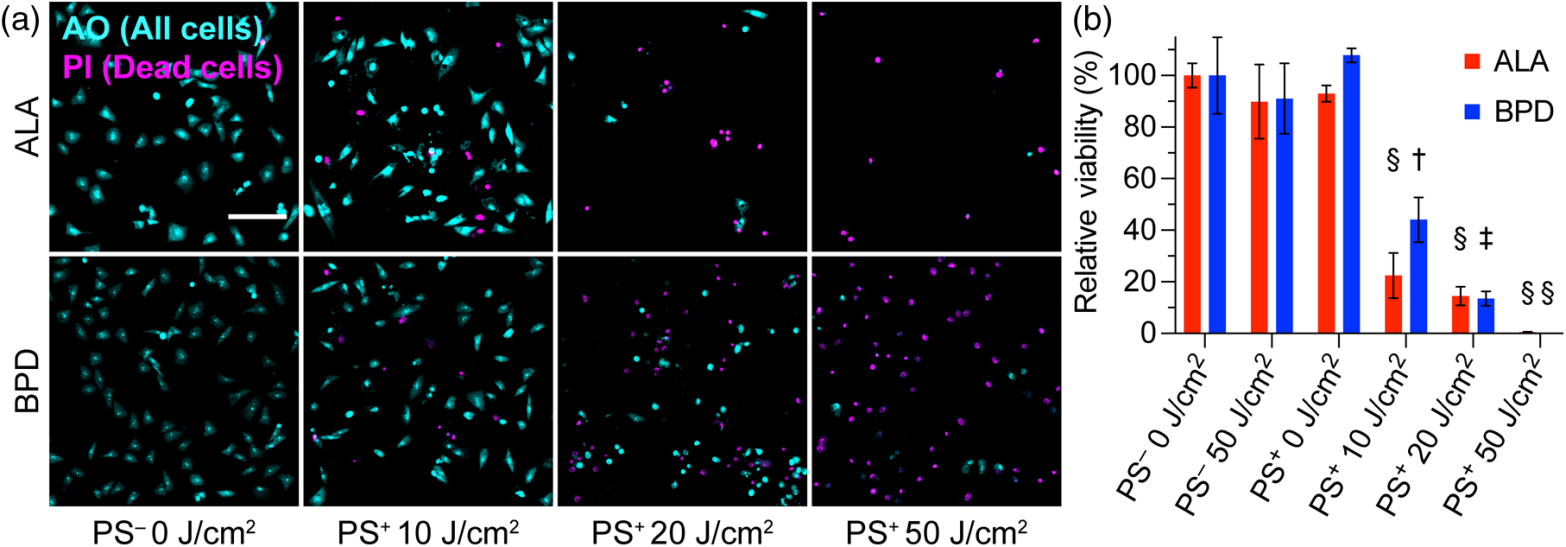
Viability of powder cells 24-h post-LED-PDT. (a) AO/PI staining shows PDT-induced cell death. Full images are provided in Fig. S5 in the Supplementary Material. Scale bar: 200  μm. (b) Relative viability±SE assesed by live/dead FC staining (n=3 biological replicates) confirms a light dose-dependant response to treatment. One-way ANOVA for each experiment was significant (p<0.0001) and Dunnett’s multiple comparisons test confirmed siginficant differences compared to the control group. ^†^p=0.0025, ^‡^p=0.0002, and ^§^p<0.0001. p-values are adjusted for multiple comparisons.

A primary ovarian cancer cell line was successfully treated with ALA- and BPD-PDT using the 635- and 690-nm modular LED arrays, respectively. No statistically significant effects were observed across control groups in both cases, but a clear, light-dependent decrease in cell viability was observed via fluorescence microscopy and quantified with live/dead FC analysis (Fig. 6). Compared to the no-light, no-PS control, $10\;{J/\mathrm{cm}}^{2}$ was sufficient to eliminate >50% of cells using each PS. At the maximum $50\;{J/\mathrm{cm}}^{2}$ dose, cell viability was 0.52±0.07% (p<0.0001) after ALA-PDT and 0.07±0.00% (p<0.0001) after BPD-PDT. Confocal microscopy of treated cells with AO/PI staining is qualitatively consistent with the dose-response trend. Full images are provided in Fig. S5 in the Supplementary Material.

## 4 Discussion

This work describes a benchtop PDT device to facilitate economical multi-PS PDT research. The device incorporates a modular design built around high-power surface-mounted LEDs. Due to the significant heat generated by such LEDs, an aluminum PCB, heat sink, and active air cooling were incorporated to maximize heat flow away from the LEDs. With active cooling, the module temperature (and therefore power) can be operated at stable equilibrium with <1% fluctuation over the course of PDT. The system was successfully used to treat a monolayer ovarian cancer model with both ALA and verteporfin wherein both experiments were performed consecutively, thus confirming its multi-PS versatility and practicality.

Beyond the present configuration, the device allows for flexibility in experimental design in many aspects. First, the modular design allows experimenters to add additional wavelengths quickly and at very low cost (∼\$150 per module, plus, excluding the computer, power meter, and 3D printer, ∼\$2000 of supporting materials as a one-time investment). This provides a significant advantage to investigators developing and testing new PSs for which commercial light sources would be prohibitively costly (e.g., a laser) or do not exist. The simple swapping of modules allows activation of different PSs within minutes, opening the door for development of combination PDT^41^^–^^43^ (as has been done extensively with chemotherapy). Additionally, the digital shutter allows for implementation of complex fractionated^6^^,^^44^^,^^45^ and metronomic^46^ phototherapeutic strategies.

Second, the large spot size (∼4×4  cm) provides for flexible use of different well plates for various applications. Here, we designed a fixed aperture to illuminate a 2×2-well group in a 24-well plate for simultaneous treatment of four biological replicates. In principle, other microplate sizes or dishes could be illuminated with varying sizes and groupings, or the aperture could be constricted to single-well illumination. Although a reduced spot size would provide a larger average power across the well, this would also increase the number of trials per plate and therefore the total plate illumination time. This is one limitation of laser-based systems we set out to overcome. Experimenters should take care to keep total plate treatment times short to avoid significant auxiliary cell death. This flexibility in well-plate design is programmed into the ExpressPDT software to allow for a custom grid of desired light doses per plate.

Finally, extension of this protocol to *in vivo* work is also feasible, as the plexiglass surface is suitable to support small animal models. For example, subcutaneous tumors in a mouse model could be epi-illuminated using the described configuration. In this work, we show that a clinically relevant $50\;{J/\mathrm{cm}}^{2}$ dose is practical and was enough to achieve >90% cell death in monolayer cell culture with multiple PSs. For large tumors, doses as high as $200\;{J/\mathrm{cm}}^{2}$ are attainable (∼32  min per dose at peak power). Of course, tissue oxygen becomes an important factor for *in vivo* PDT^6^ and doses larger than 40 to $50\;{J/\mathrm{cm}}^{2}$ present with diminishing therapeutic returns.^13^ As mentioned above, an automated fractionation protocol becomes an important component of high-dose PDT, which may be developed and tested both *in vitro* and *in vivo* using this device.

Further optimizations would improve the device for future implementations. First, including a photodiode would allow for automated power monitoring after calibration similar to the method for thermistor calibration described herein. Second, denser arrays with a larger footprint could be designed to provide a more uniform spot size for larger treatment groups or even whole-plate illumination. Third, off-the-shelf water-cooling systems for high-power computer CPUs could also be adopted to provide a more efficient cooling system for the board, allowing the LEDs to operate at higher powers and reducing the risk of thermal damage to the board or its components. However, water cooling may be undesirable for a mobile-cart implementation. Finally, an investigation into the lifespan of the LED module was not done here but may be warranted for instances of high-throughput use.

In summary, we devised a versatile and cost-effective device to enable and improve the forefront of PDT research. Design of the LED module for efficient heat transfer enabled stable, high-power output, which was used as an effective treatment of a primary ovarian cancer model *in vitro*. The modular design begets a low-cost-per-wavelength device to facilitate next-generation, multi-PS research, which is not practical with existing commercial light sources. Additionally, a custom application, ExpressPDT, is included for streamlined experimental planning and semiautomated protocol implementation. This work is intended to aid the research community in developing the next generation of phototherapy and PDT using LEDs as valuable and versatile light source.
