# Supplementary material for: High-power light-emitting diode array design and assembly for practical photodynamic therapy research
Source: J Biomed Opt. 2020 Apr 15;25(6):063811. doi: 10.1117/1.JBO.25.6.063811 (PMC7156854; doi:10.1117/1.JBO.25.6.063811)
Supplement: Supplementary file 1 [file JBO_025_063811_SD001.pdf]

Supplementary Information

High-power LED array design and assembly for practical photodynamic therapy research

Eric M Kercher, Kai Zhang, Matt Waguespack, Ryan T Lang, Alejandro Olmos, Bryan Q Spring*

***Corresponding Author**, E-mail: b.spring@northeastern.edu

This file contains:

Figures S1–S5

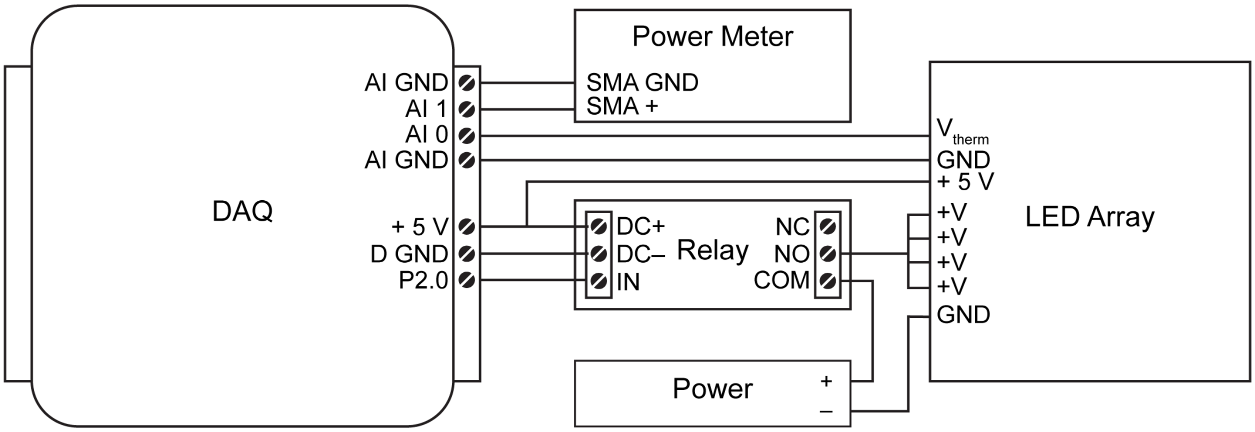

Figure S1. Wiring Diagram for the LED Array experimental setup. The LED Array module is powered by a 30V/5A power supply with a relay that is digitally controlled via data acquisition card (DAQ). The DAQ is also used to read voltage signal from the LED module thermistor (V_{therm}) and compatible power meter. The DAQ is connected to a computer via USB–USB-c cable (not shown). Devices not drawn to scale.

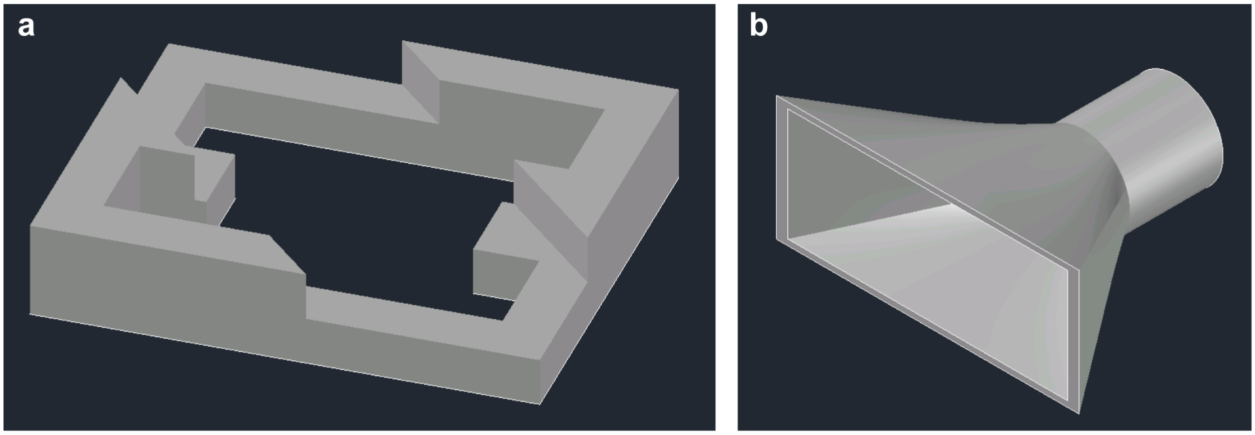

Figure S2. Custom 3D-printed parts for LED-PDT experimentation. (**a**) A brace designed to fit around a vertical cage mounting system (CPVM, Thorlabs). The module slides into the brace (between two support posts) at a 45-degree angle to restrict movement in one planar dimension. (**b**) Nozzle for directing air flow from an air line through the LED module heat sink fins.

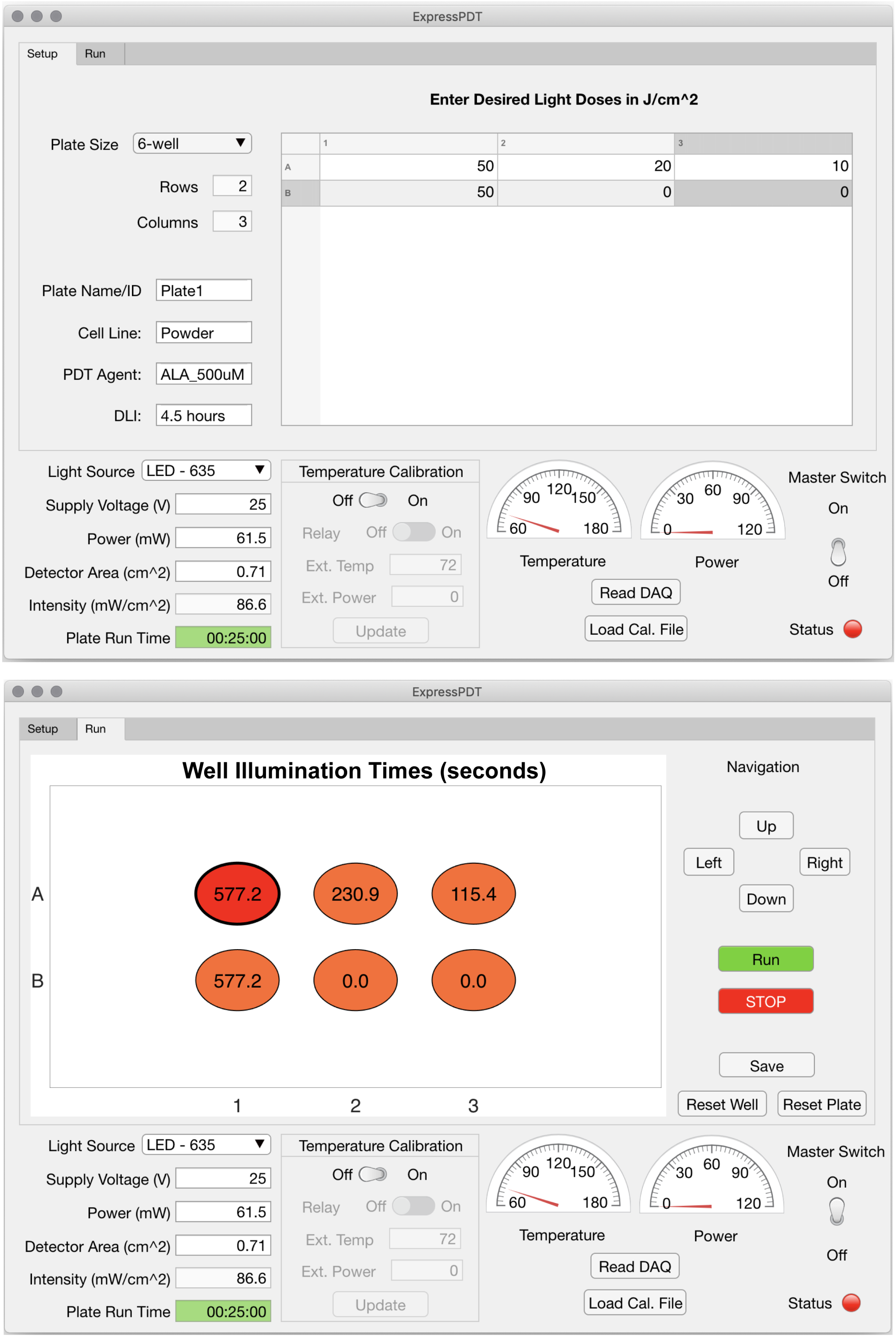

Figure S3. ExpressPDT Software written using App Designer (MATLAB, R2019b). (Top) Well plate illumination planning occurs by adjusting the Plate Size to match the group dimensions and entering the desired light doses in the table. (Bottom) The user selects each group using the

navigation panel and a countdown is animated while the shutter is open. Code is available at www.springlabnu.com/software.

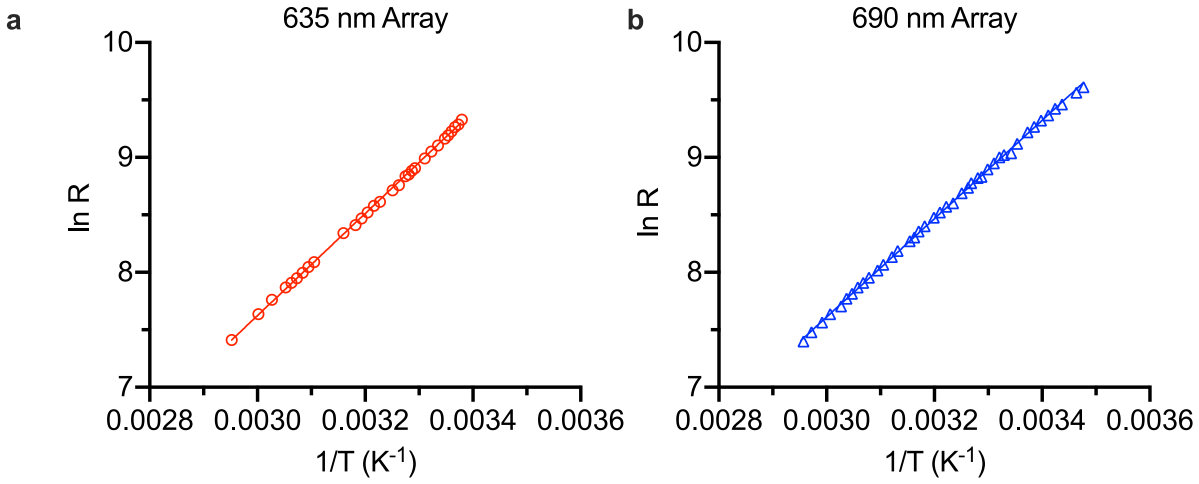

Figure S4. Temperature and thermistor voltage data from Fig. **2** was linearized according to eq. (3) and fit via linear regression with slope β and intercept ln R_∞. (**a**) 635 nm module parameters are β = 4442 ± 16 K and ln R_∞ = –5.70 ± 0.05, R^2 = 0.999. (**b**) 690 nm module best fit values are β = 4279 ± 7 K and ln R_∞ = -5.23 ± 0.03, R^2 = 0.999. Intercepts are converted to R_∞ in mΩ in the text.

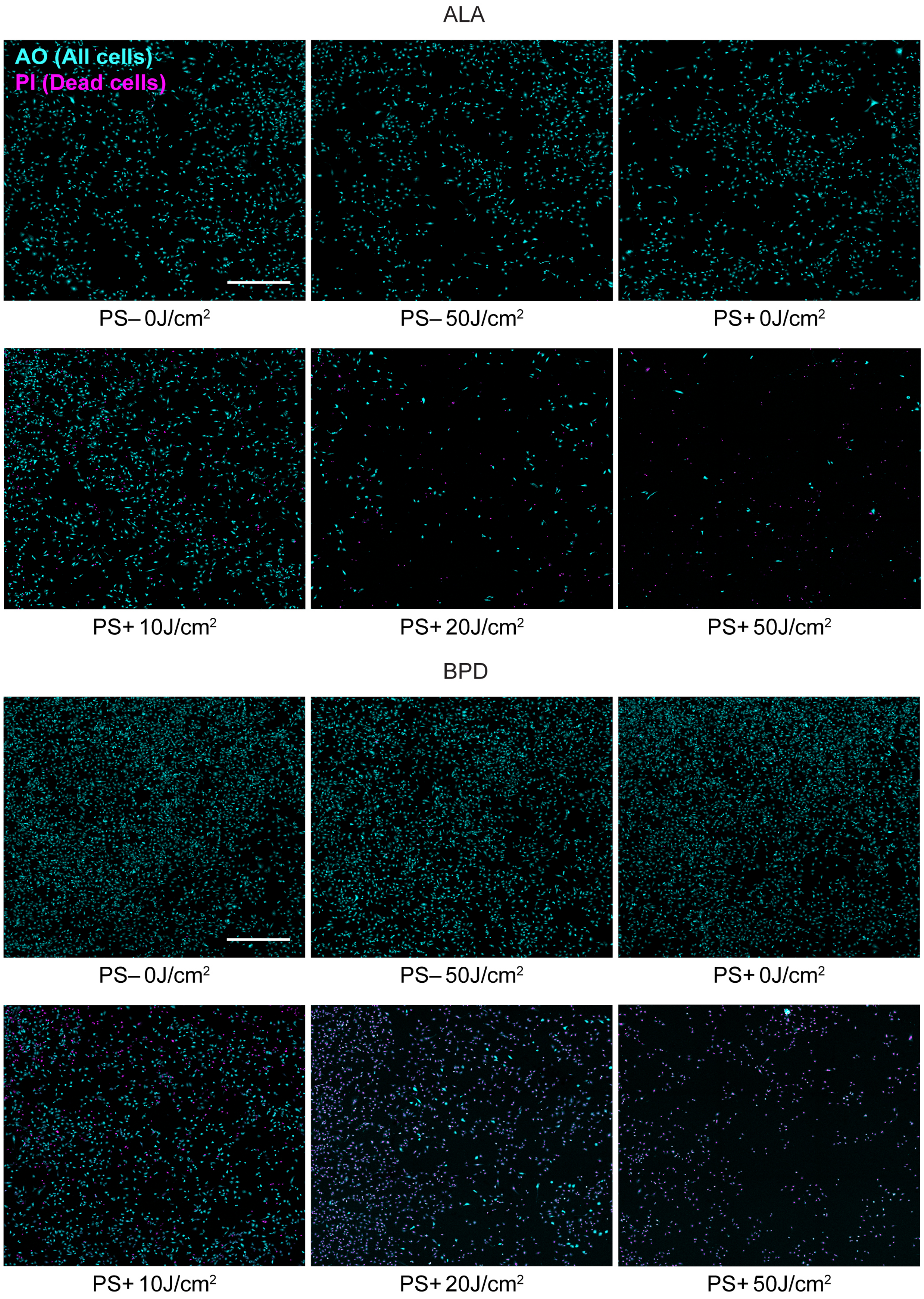

Figure S5. Live/Dead confocal microscopy 24 hours post LED-PDT. Scale bar, 1 mm.
